# Supplementary material for: Complexome profiling of the Chlamydomonas psb28 mutant reveals TEF5 as an early PSII assembly factor
Source: Plant Cell. 2025 Mar 17;37(6):koaf055. doi: 10.1093/plcell/koaf055 (PMC12164590; doi:10.1093/plcell/koaf055)
Supplement: koaf055_Supplementary_Data [file koaf055_supplementary_data.zip › psb28_interactive.zip!/plots/intensity/qualityPlots/peptides/AAT1_Cre10.g451950.t1.2.html]

### Cre10.g451950.t1.2

Trivial name: AAT1  
SeqCoverage [median]: 58.20  
Mass: 57.82 kDa  
  
 

| Sequence | MissedCleavage | Mass [Da] | wtIntensity | muIntensity | Score |
| --- | --- | --- | --- | --- | --- |
| ALQEAREEGK | 1 | 1129.57 | 18756.81 | 24216.34 | 123.96 |
| ALQEAREEGKLVR | 2 | 1497.83 | 3784.82 | 9654.24 | 105.65 |
| ASELVSLR | 0 | 873.49 | 24892.44 | 39228.60 | 170.66 |
| ASELVSLRR | 1 | 1029.59 | 0.00 | 1412.09 | 93.43 |
| DGVPSNPDHIFLTDGASVAVR | 0 | 2166.07 | 2145.81 | 14675.12 | 81.67 |
| EEGKLVR | 1 | 829.47 | 0.00 | 657.18 | 116.30 |
| EEVMTHFVEK | 0 | 1247.59 | 9198.18 | 18872.15 | 120.15 |
| EGKEIIFTNVGNPHALGAK | 1 | 1994.06 | 0.00 | 1164.77 | 46.25 |
| EIIFTNVGNPHALGAK | 0 | 1679.90 | 295.07 | 15612.76 | 85.42 |
| EIIFTNVGNPHALGAKPLTFTR | 1 | 2395.30 | 4428.61 | 26478.70 | 101.94 |
| EKASELVSLR | 1 | 1130.63 | 0.00 | 1706.90 | 67.70 |
| ENLQELIK | 0 | 985.54 | 3017.85 | 14582.56 | 136.21 |
| FAYQEK | 0 | 784.38 | 7519.22 | 4316.82 | 117.70 |
| GELYLR | 0 | 749.41 | 3057.90 | 1623.93 | 109.94 |
| GGVGAYTDSR | 0 | 981.45 | 9220.51 | 20175.01 | 107.57 |
| GGYVEMTNIHPGAIEEVYK | 0 | 2106.01 | 1279.40 | 6068.79 | 78.95 |
| GNPLVREEVAR | 1 | 1238.67 | 5704.89 | 9597.51 | 69.98 |
| GWGLSVEELQR | 0 | 1272.65 | 0.00 | 1567.06 | 116.52 |
| HDRDSVLVPIPQYPLYSASIR | 1 | 2425.28 | 811.57 | 0.00 | 41.26 |
| ILASFKGGVGAYTDSR | 1 | 1640.85 | 0.00 | 1870.27 | 32.81 |
| IVLMADEVYQENVYQDERPFVSAK | 1 | 2842.38 | 5362.18 | 11015.37 | 127.01 |
| KVMWEMGEPYR | 1 | 1424.66 | 1293.85 | 2924.58 | 74.96 |
| LLEATGISTVPGSGFGQEEGTFHLR | 0 | 2602.30 | 5711.45 | 0.00 | 82.59 |
| LYGGTLVGYFLDER | 0 | 1601.81 | 5290.50 | 0.00 | 142.10 |
| RGWGLSVEELQR | 1 | 1428.75 | 0.00 | 7843.34 | 82.54 |
| SHVELLSFHTVSK | 0 | 1482.78 | 2654.28 | 35611.93 | 152.47 |
| TQYAVR | 0 | 736.39 | 2805.28 | 14272.19 | 118.69 |
| TTILPR | 0 | 699.43 | 9481.70 | 13888.20 | 130.84 |
| TTILPREEVMTHFVEK | 1 | 1929.00 | 0.00 | 2448.42 | 64.39 |
| VEDMFPADAIAR | 0 | 1333.63 | 650.91 | 3583.66 | 107.83 |
| VLHPHLLNENVVK | 0 | 1510.86 | 4616.26 | 29034.84 | 184.31 |
|  |  |  | 131979.5 | 334103.3 |  |
